# Qualifying and quantifying the precision medicine rhetoric

**DOI:** 10.1186/s12864-019-6242-8

**Published:** 2019-11-15

**Authors:** Jasmine Lee, Dina Hamideh, Camille Nebeker

**Affiliations:** 1 Case Western Reserve University, Cleveland, OH 44106 USA; 2 The Scripps Research Institute, La Jolla, CA 92037 USA; 3 Department of Family Medicine and Public Health, School of Medicine, UC San Diego, La Jolla, CA 92093-0811 USA

**Keywords:** Precision medicine, Personalized medicine, Genomic medicine, Individualized medicine, Evidence-based medicine, Stratified medicine, Big Data, Translational medicine, P4 medicine

## Abstract

**Background:**

With the rise of precision medicine efforts worldwide, our study objective was to describe and map the emerging precision medicine landscape. A Google search was conducted between June 19, 2017 to July 20, 2017 to examine how “precision medicine” and its analogous terminology were used to describe precision medicine efforts. Resulting web-pages were reviewed for geographic location, data type(s), program aim(s), sample size, duration, and the key search terms used and recorded in a database. Descriptive statistics were applied to quantify terminology used to describe specific precision medicine efforts. Qualitative data were analyzed for content and patterns.

**Results:**

Of the 108 programs identified through our search, 84% collected only biospecimen(s) and, of those that collected at least two data types, 42% mentioned both Electronic Health Records (EHR) and biospecimen. Given the majority of efforts limited to biospecimen(s) use, genetic research seems to be prioritized in association with precision medicine. Roughly, 54% were found to collect two or more data types, which limits the output of information that may contribute to understanding of the interplay of genetic, lifestyle, and environmental factors. Over half were government-funded with roughly a third being industry-funded. Most initiatives were concentrated in the United States, Europe, and Asia.

**Conclusions:**

To our knowledge, this is the first study to map and qualify the global precision medicine landscape. Our findings reveal that precision medicine efforts range from large model cohort studies involving multidimensional, longitudinal data to biorepositories with a collection of blood samples. We present a spectrum where past, present, and future PM-like efforts can fall based on their scope and potential impact. If precision medicine is based on genes, lifestyle and environmental factors, we recommend programs claiming to be precision medicine initiatives to incorporate multidimensional data that can inform a holistic approach to healthcare.

## Background

Precision medicine promises tailored treatments and health promotion strategies based on an individual’s biology, environment, and lifestyle choices [1, 2]. In 2015, President Obama announced the Precision Medicine Initiative (PMI), a research agenda to explore the genetics of cancer, improve access to personalized health information, and collect data from a diverse national cohort [3]. A major component of the PMI is the National Institutes of Health’s (NIH) *All of Us* Research Program (AoURP), which seeks to create a research repository consisting of biospecimens, electronic health records (EHRs), physical measurements, and lifestyle data from one million U.S. participants in order to glean causes and cures for various diseases [4]. The AoURP’s goal to study individual and social determinants of health on a large scale is not unique. It falls in line with past seminal research studies that sought to identify risk factors for complex diseases by collecting civilian health data longitudinally, such as the pioneer Framingham Heart Study (1948) and the Nurses’ Health Study (1976), which contributed invaluably to our understanding of cardiovascular and reproductive health, respectively [5, 6]. What makes the AoURP distinct is its temporality. It is capitalizing on emerging technologies such as high-throughput sequencing, wearable biosensors, and the Internet to collect and share new types of health data. With the integration of new and more data, the promise of precision medicine has led to a plethora of programs that involve varied objectives, processes, and stakeholders. Perhaps these efforts will improve our existing healthcare system yet, in the meantime, the broad use of the precision medicine label may breed confusion and delay progress.

In 2011, the National Research Council (NRC) formally introduced “precision medicine” as requiring a centralized, widely accessible database that will afford a “new taxonomy” of disease [2]. The NRC argued for it to supersede “personalized medicine,” which can be confused for the design of unique, rather than more precise and accurate, interventions for each patient. There have since been numerous debates about the scope of precision medicine. Scholars cite its misuse when describing only the use of genomics in clinical care [7]; its foundations in translational medicine [8]; its reconciliation with evidence-based medicine, where N-of-one cases have to be corroborated through large-scale databases [9]; its deferral from population health and social policy, which may be mediated by “precision public health;” [10, 11] and its undue focus on “genomic and molecular discovery” as opposed to electronic health records and data analytics [12]. There seems to be a clear demarcation between precision medicine and genomic medicine in that the former is dynamic, incorporating behavioral and environmental influences in addition to genomes, phenotypes, and clinical data [7, 13]. One can begin to gauge how complex the general terminology is when describing an effort branded as precision medicine.

Initiatives are typically considered the first steps of something greater, akin to moonshots [14]. There is concern that programs using the label “precision medicine (initiative)” are exploiting the excitement or buzz around efforts that are more comprehensive, which can perpetuate misconceptions by consumers – patients, practitioners, and researchers alike. Thoughtful communications are needed to ensure feasibility of seemingly infinite sources of health data and to reduce misinformation to the public. The importance of consistent nomenclature may not be fully realized until preventable problems occur. For example, when patients enroll in a “precision medicine” study, do they know who has perpetual access to their data, what research their data will be used towards, and whether they have a right to their results? Unless dutifully informed, they may feel deceived or blindsided, reluctant to continue and/or contribute to future studies. Public trust and autonomy are especially important in the recruitment of study subjects today because, over the past decade, there has been an increasing shift in their perceived role. Increasingly, they are becoming active participants who can provide insight on research questions and study design [1, 15]. When well-informed and engaged, participants can also best advocate for the sharing of their personal health data to both garner public support and safeguard privacy [16, 17]. Therefore, we sought to better understand the scope of precision medicine and explore trends in its advertisement to the public by compiling and delineating programs that use “precision medicine” phrasing. A catalogue of the precision medicine ecosystem would help to not only map the scope and breadth of programs, but also to standardize practices for obtaining consent and risk management protocols in order to foster ethical research and scientific integrity. Moreover, today research studies are more likely to include multiple recruitment sites with ongoing health data donations, so defining categories of research can help match the level of oversight with the level of risk posed [18].

Efforts to define and map such an ecosystem include a list of global “genomic medicine” studies and a national network of collaborators in “precision medicine” in the United Kingdom [19–21]. However, a global inventory of “precision medicine” programs based on common search terms and a holistic, public-oriented perspective does not exist. This paper describes steps taken to quantify and qualify programs, studies, or organizations identified via search terms synonymous with precision medicine. By documenting past and current studies that identify as precision medicine, we can document the scope of programs and subsequently educate the public to increase awareness and manage expectations.

## Results

**Fig. 1:**
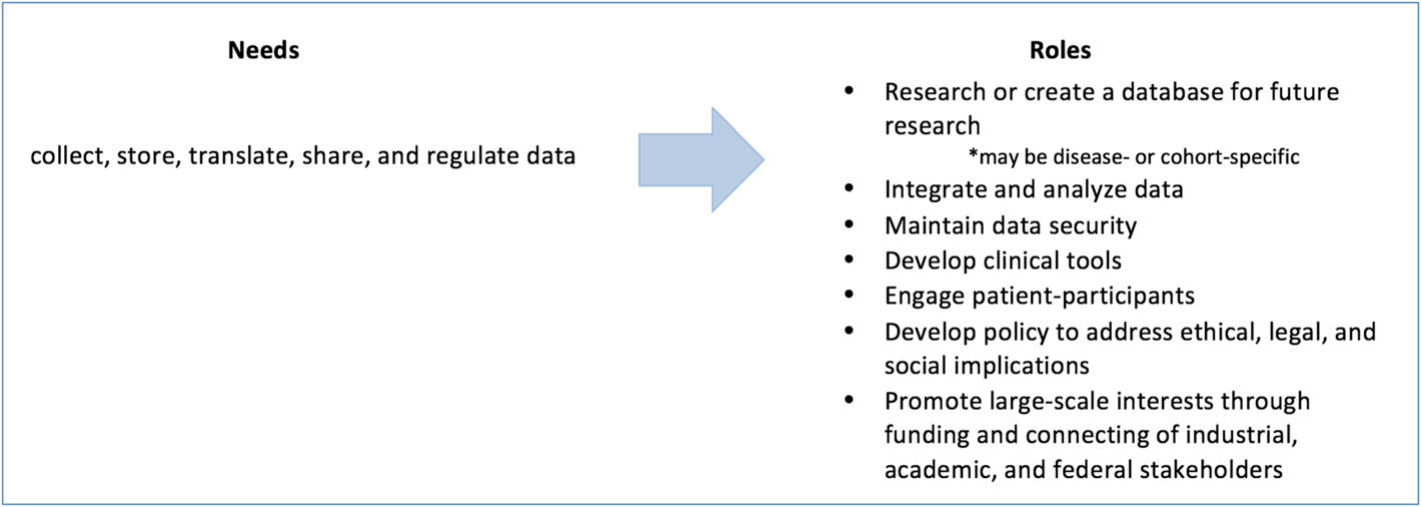
An online, exploratory search of precision medicine efforts revealed multiple needs revolving around data that warrant diverse roles

The search resulted in 108 programs or studies that used one or more of the key search terms on their webpage. Extrapolating from our qualitative data with regard for the program’s aim, the following themes specific to functions and roles in the field were identified (see Fig. 1). The function of precision medicine involves paving ways to collect, store, translate, share, and regulate ever-increasing amounts of health data. These actions in turn call on researchers, industries, governments, and other organizations to create baseline databases, effectively and safely compile the data, translate the data and develop applicable tools, and more.

### Global distribution of precision medicine efforts

**Fig. 2:**
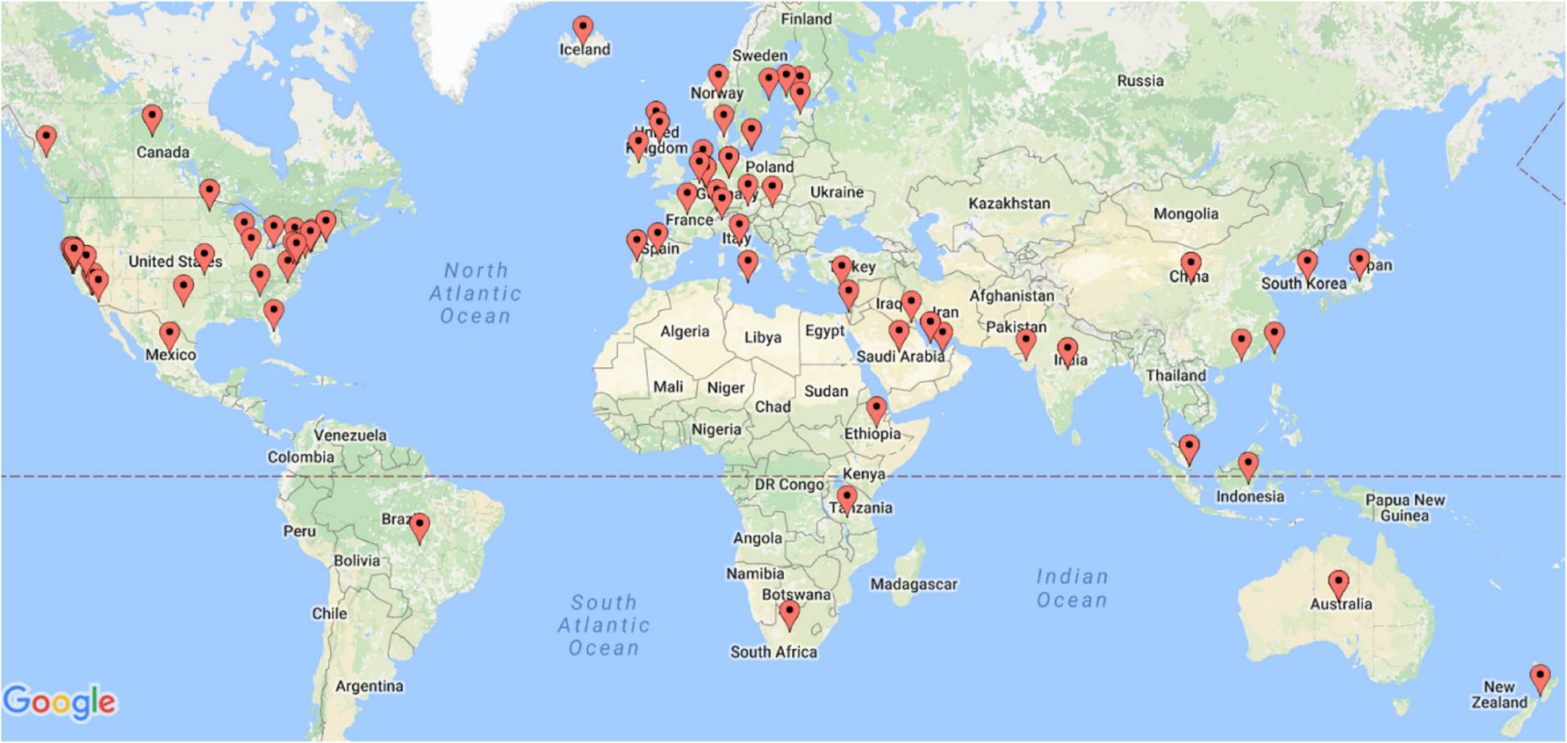
Global precision medicine efforts identified using search terms from Table I. The Google Map was generated through BatchGeo, an open source mapping tool

The global distribution of PM-like efforts shows the widespread publicizing of precision medicine (see Fig. 2). Although 87 unique countries were included in the search syntax, one-third of the programs identified (*n* = 36) were found to be domestic with its host institution or cohort in the United States. Initiatives linked to “precision medicine” were concentrated in the United States, Europe, and the Asian countries with the highest gross domestic product (GDP). While this result may be biased based on our search method, the list of the fifty most populous countries did not yield nearly as many PM-like efforts (*n* = 19) as the list of the fifty wealthiest countries did (*n* = 33). Furthermore, out of the 13 countries that present on both lists as being both wealthy and populated, ten were linked to a PM-like effort: United States, Saudi Arabia, Germany, Taiwan, Canada, United Kingdom, France, Japan, South Korea, and Italy.

### Trends in purpose, data sources, funding, and study criterion

Statistics on the characterization of the PM-like efforts are summarized in Table 1. Forty-three percent (*n* = 46) were unidimensional whereby they mentioned collecting only one of the data types listed and/or only working with existing databases, and 54% (*n* = 58) collected at least two data types. Existing databases refer to genomic and/or clinical datasets that are not new or ongoing. While a study may be using multivariable data, the data collection is still unidimensional. Four studies were unclear about data source(s). Eighty-four percent (*n* = 91) collected biospecimen(s), and 42% (*n* = 45) involved Electronic Health Records (EHR). The large majority of studies classified as unidimensional were specific to biological data – 33 of the 46 unidimensional studies (72%) were only explicit about biospecimen(s) collection, excepting the integration of existing databases. Of the studies that specified collecting two or more data types (i.e. multidimensional studies), 72% (*n* = 42) combined results from biospecimen(s) with EHR. Person provided information (PPI), such as patient self-report surveys and behavior questionnaires, and sensor data were far less common in our database. Twenty-four percent (*n* = 26) of the programs used PPI, and 10% (*n* = 11) used sensor data.

**Table 1:** Descriptive Categories Used for Statistical Analysis

Theme	Category	Number of PM-like efforts	Percentage of total
Location *of funding institution*	Domestic	36	33.3%
	Global	72	66.7%
Longitudinal		22	20.2%
Sample size	*≥* 10,000	19	17.6%
	< 10,000	16	14.8%
	Unknown	70	67.6%
Data types	Unidimensional	46	42.6%
	Two or more	58	53.7%
	Unclear	4	3.7%
	Biospecimen	91	84.3%
	Biospecimen only	35	32.4%
	EHR	45	41.7%
	PPI	26	24.0%
	Sensor	11	10.2%
Duration	Explicit	17	15.7%
Participant as partners	Yes	25	23.1%
	No	25	23.1%
	Unknown	58	53.7%
Study Aim	Database	69	63.9%
	Clinical Trial	44	40.7%
Funding	Government	59	54.6%
	Industry	35	32.4%
	Nonprofit	12	11.1%
	Donations	7	6.5%

Of the 108 programs identified, 18% (*n* = 19) intend to enroll at least 10,000 people, the highest denominator belonging to the partnership between Yale University and the National Center for Cardiovascular Disease in China with a target population of 4 million [22]. Most of the studies that were explicit about sample size were cohort models designed to create a research resource from which scientists could later access the data to answer more specific questions (*n* = 30). Based on our classification of the program aims, 64% (*n* = 69) functioned in part as a database, where 23% (*n* = 25) functioned exclusively as a database. Forty-one percent (*n* = 44) ran clinical trials. As such, we can speculate that most “precision medicine” endeavors currently emphasize the foundational or ongoing collection of data, and to a lesser degree, clinical intervention. No “N-of-1” studies were found, which may speak to the clarity that the National Research Council had provided in preferring the term “precision medicine” over tailored, “personalized” efforts with its 2011 report.

**Fig. 3:**
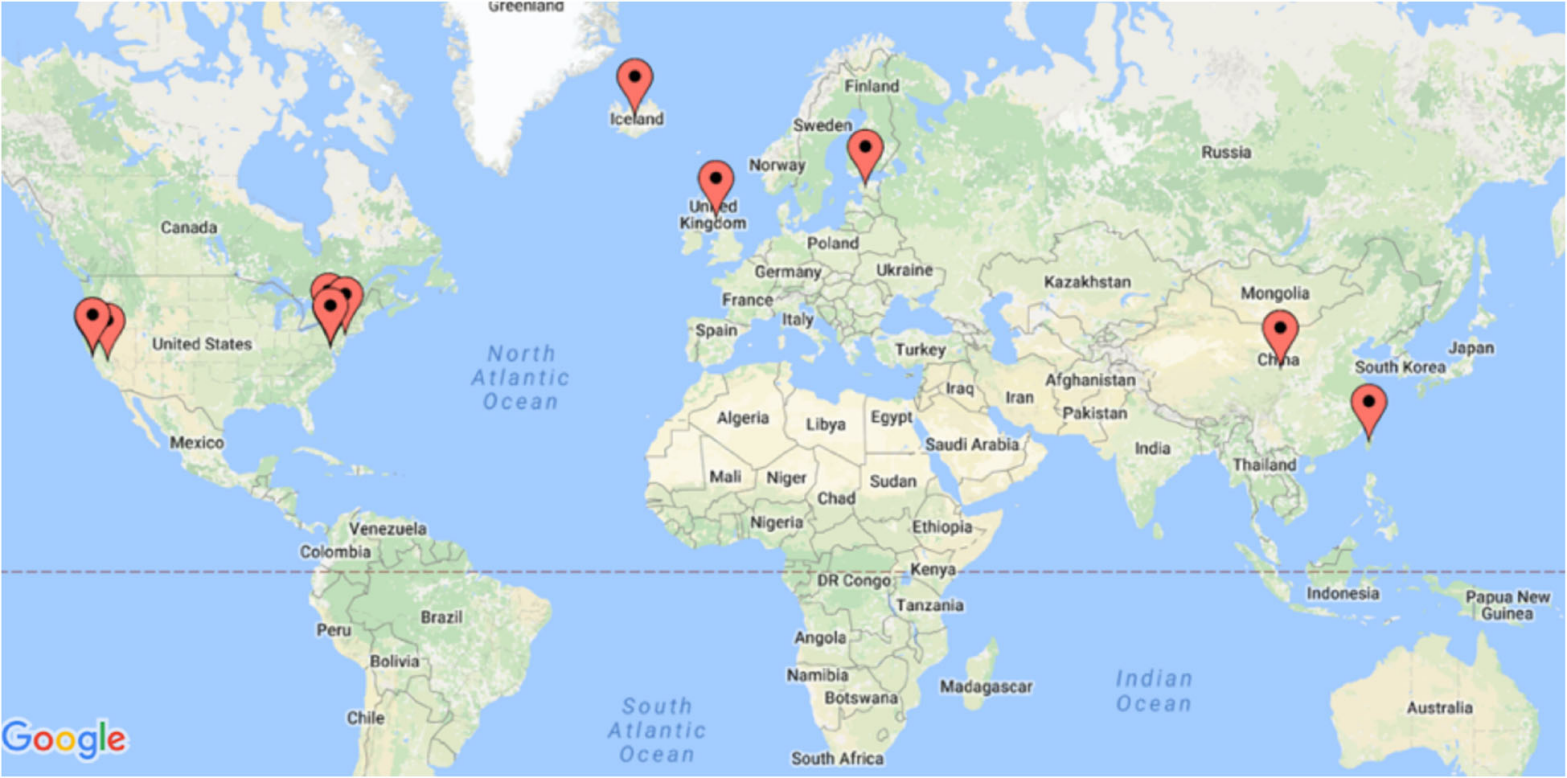
Longitudinal global precision medicine efforts collecting at least two data sources involving ≥10,000 participants as of June through July 2017. Note: Some points overlap due to the lack of a unique location given besides country. The Google Map was generated through BatchGeo

Moreover, of the 108 programs, 20% (*n* = 22) were longitudinal, and 23% (*n* = 25) used verbiage describing participants as partners. For example, InnVentis’ mission to “empower patients, payers and providers with actionable insights in real time,” “enabling the patient to self-monitor their personal precision treatment” depicts how patient interests and involvement is front and center [23]. Furthermore, 55% (*n* = 59) obtained funding through a government entity and 32% (*n* = 35) through industry support, while 14 programs (13%) indicated a government-industry partnership. This large proportion of industry and government stakeholders suggests that PM-like efforts tend to be top-down, requiring substantial resources and coordination between multiple disciplines and entities (e.g., hospitals, biotechnology and data science companies, research institutions, and ethics committees). Lastly, only 13% (*n* = 14) resembled the scope of large-scale initiatives similar to the NIH’s AoURP and Grail, meaning they sought to involve at least 10,000 people, collect at least two data sources, and were longitudinal (see Table 2 and Fig. 3).

**Table 2:** Longitudinal Programs Collecting ≥ 2 Data Types with a Sample Size ≥ 10 K

Study Name	Location	Data types	Minimum Sample Size	Duration
All of Us Research Program	USA	Biospecimen, EHR, PPI, Sensor	1,000,000	10 years
The Human Project of NYU	USA (NYC)	Biospecimen, EHR, PPI, Sensor	10,000	20 years
Project Baseline	USA	Biospecimen, EHR, PPI, Sensor	10,000	10 years
100,000 Genomes Project	UK	Biospecimen, EHR, PPI	75,000	5 years
Research Program on Genes, Environment, and Health (RPGEH)	USA	Biospecimen, EHR, PPI	500,000	–
PEACE Millions Persons Project	China	Biospecimen, PPI	4,000,000	–
Estonian Program from Personal Medicine	Estonia	Biospecimen, EHR, PPI	500,000	–
GRAIL, Inc.	USA	Biospecimen, EHR	120,000	5 years
Million Veteran Program (MVP)	USA	Biospecimen, EHR, PPI	1,000,000	5 years
DiscovEHR Collaboration	USA	Biospecimen, EHR	50,762	–
Chinese Precision Medicine Initiative	China	Biospecimen, Sensor	1,000,000	15 years
Taiwan Biobank	Taiwan	Biospecimen, EHR, PPI	300,000	10 years
Iceland Genome Project (DeCODE)	Iceland	Biospecimen, EHR	10,000	–
10,000 Families Study at the University of Minnesota	USA	Biospecimen, EHR, PPI	10,000	–

**Fig. 4:**
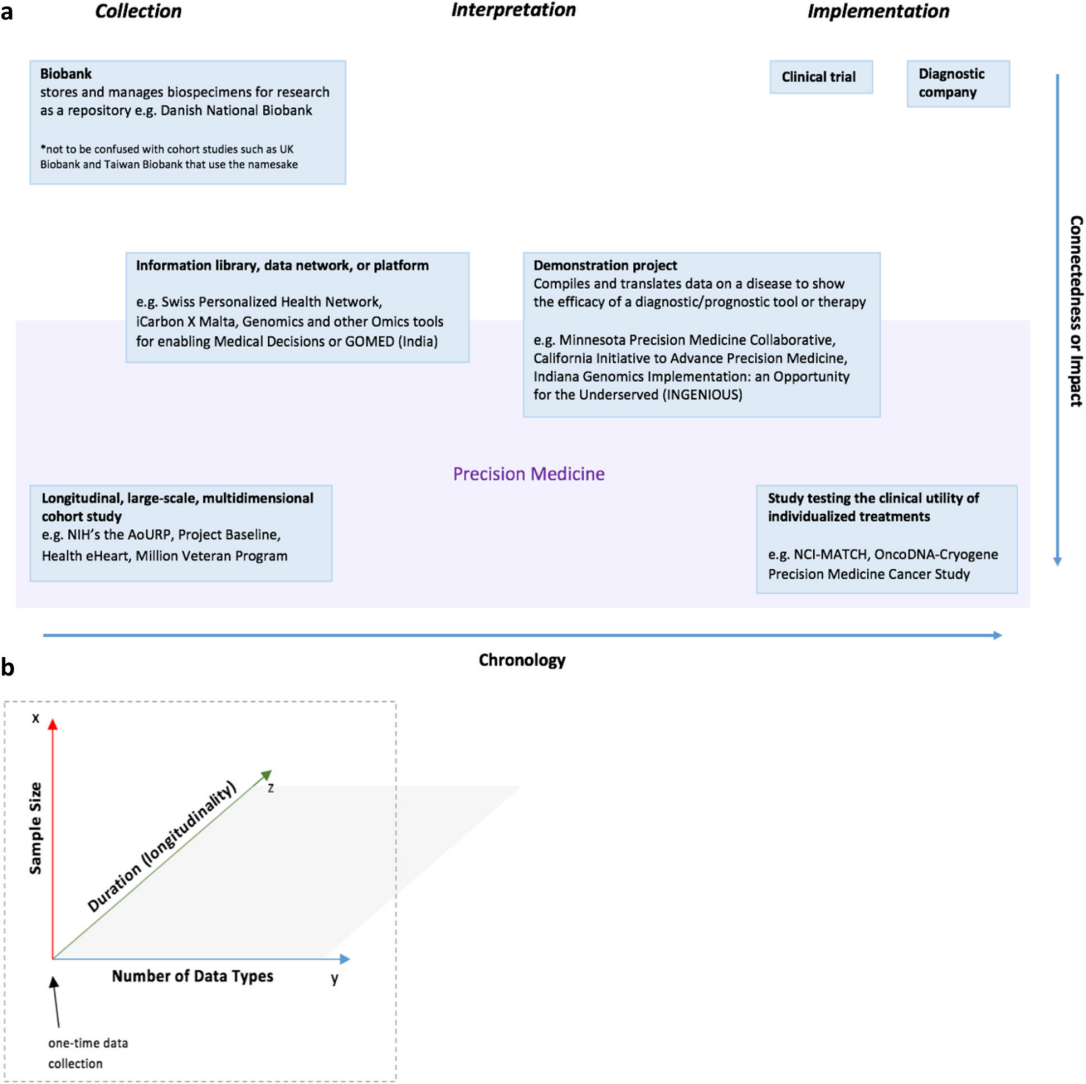
**a** The precision medicine spectrum was theorized based on PM-like efforts found using its rhetoric. A program, study, or consortium may have greater impact by being multidisciplinary and establishing connections across different stages of data acquisition and use. **b** The collection of data by a particular study can be further stratified according to dimensions of sample size, longitudinal design (and if positive, duration), and number of data types. *The data presented* e.g. *general roles and specific program names, were extrapolated and pulled from our qualitative database (n = 108)*

## Discussion

Our research revealed a broad range of programs using “precision medicine” branding and its analogous terms, including genomic data repositories, demonstration projects, and diagnostic companies. The majority of programs involved biospecimen collection, which underscores the prevalence of genetic testing, drug targeting, and biopsy. This points to the heavy influence of genetics and genomics in precision medicine, in part due to such data being readily available and interpretable [24]. The main approaches to genomic medicine gathered from the 2014 Global Leaders in Genomic Medicine symposium were well observed throughout our search: integration of genomic sequencing with EHR, national research programs, and highly focused pilot studies to build capacity and show efficacy [20]. Specifically, more than a third of our database (*n* = 42) plan to integrate biospecimen results with EHR. Barriers to the use of genetic data in routine clinical care include lack of evidence of clinical utility, standardization of practice, and in-hospital informatics infrastructure, [20] but the growing number of studies that combine these two data types are addressing these issues if not demanding future work on them. The variety of identified efforts also targeted the concerns that the WISH Precision Medicine Forum (2016) had proposed as key action areas for the sustainability of precision medicine: evidence generation, patient and public engagement, implementation, and data privacy and sharing [19].

Based on our findings, “precision medicine” and its analogous terms are used to describe programs that are diverse in scope – from biobanks, translational facilities, and clinical trials to national, community-wide, and disease-specific cohort studies. Yet, our search conceived a spectrum chronicaling the collection, interpretation, and implementation of data in precision medicine (see Fig. 4). It also revealed a small subset of programs that share the purpose of forming a transdisciplinary network, gathering multidimensional data, and making the data available for future research. Such programs, including the AoURP, Project Baseline, and Taiwan Biobank, underscore the importance of a large cohort; numerous determinants of health; and longitudinal research where participants may become partners in the endeavor. Table II contains domestic and global programs found from our query that align with this subcategory, which we argue represent precision medicine initiatives. Large precision medicine cohort initiatives are being fostered by “recent advances in genome sequencing, cohort study designs, health informatics, and wireless technologies” that, together, can shine light on the genetic, behavioral and environmental determinants of health [13].

Our findings reveal that precision medicine exists as a spectrum of programs and consortia that seek to contribute to one or many aspects of individualized healthcare. Regardless of whether a study or program meets the ambitious goals of a precision medicine initiative, it will contribute to our understanding of the breadth of human diversity and disease. For example, there is concern over the use of “precision public health” to describe traditional studies that are receptive to genomics but are still rooted in addressing the social determinants of health [25]. A public health study should be recognized for its civic, population-based approach, but its ability to contribute to precision medicine by collecting and sharing more precise health data should not be dismissed. We argue that the colloquial definition of “precision medicine” needs to adapt to move beyond its focus on molecular discovery and technological innovation, so public health does not have its interests at stake when tied to “precision” – the opposite should be true. Moreover, we see increasing infrastructure to support *N* = 1 studies including recognition of single subject A/B testing and cross-over designs to examine unique and rare medical conditions, as well as, federal guidelines (see AHRQ [26]) and funding to support this form of precision medicine. There is clearly room in precision medicine for studies to range from an n of 1 to 1,000,000 or more. The key is to have the data necessary to understand the biological, social and environmental markers of health and illness.

The past decade has brought monumental changes in biomedical research and, no doubt the new tools that make precision medicine possible will continue to shift how health research is conducted. If precision medicine seeks to provide patients with the right healthcare at the right time, it requires research on the root causes of disease onset and on markers of its progression. Since health changes can only truly be measured over time, we wonder if more than 20% of the programs in our database that use precision medicine branding should be longitudinal by design. If precision medicine seeks to benefit the individualized healthcare of all persons, including the historically underrepresented who are most susceptible to health disparities, health data that is representative of our census should be a priority. Participants should be engaged early in the design phase as true partners, where they can advocate for the most feasible methods of data collection and corroborate the value of newfound research questions.

### Limitations

We relied on the Google search engine to identify efforts linked to precision medicine. Due to the indefinite nature of the web, our study is not truly replicable. A person may apply our criteria and obtain very similar results but also perhaps new or previously missed programs by looking into a few extra pages or clicking an extra link. However, our data was scrutinized for coding consistency, and we foremost sought to identify and bring to light (the context of) global programs prescribing to precision medicine. This research was also time-bound to 1 month in 2017 to capture a snapshot of activity and does not reflect programs that did not have a website or had poor search engine optimization scores. Furthermore, data across each website were inconsistent and based on what the site wanted to publicize. With our case study methodology, we underscore the claim that uncertainty is inherent in precision medicine as “precision is sacrificed for interpretability.” [27] That being said, we believe this is the only study that has attempted to identify and qualify the use of precision medicine as a brand presented on public facing project websites or reference articles.

## Conclusions

How behavioral and biomedical research is carried out is rapidly changing in the twenty-first century and era of precision medicine [28]. These changes introduce new challenges and opportunities including the ability to design and deploy studies that involve one to tens of thousands of people and the collection of multidimensional datasets. Our snapshot revealed that programs using “precision medicine” branding focused predominantly on genetic and genomic data. Many of the programs in our database (43%) only collected one data type, the vast majority being biospecimen(s) (72%). Only 22% were evidently longitudinal and, fewer than a quarter expressed an explicit interest in participant engagement.

While a minority, programs integrating genomic data with other health determinants, including lifestyle and environment are the pioneers as they are more likely to succeed in accomplishing precision medicine. Programs that seek to lead in the field are encouraged to develop a network of partners and engage participants as partners, such as in returning results and developing open records of current and future research projects. Moving forward, we recommend that an online catalogue or moderated database of groups identifying with “precision medicine” would be invaluable to account for roles and research interests, as well as to foster collaboration. Moreover, efforts to describe the types of omics (e.g., proteomics, radiomics) along with clinical field (e.g., oncology, cardiology) would add to our understanding of how “precision medicine” is being used in public facing media. There will always be uncertainty in precision medicine, but what makes it such a transformative endeavor is its acknowledgement of uncertainty and engagement of more people in more ways than ever before.

## Methods

**Fig. 5:**
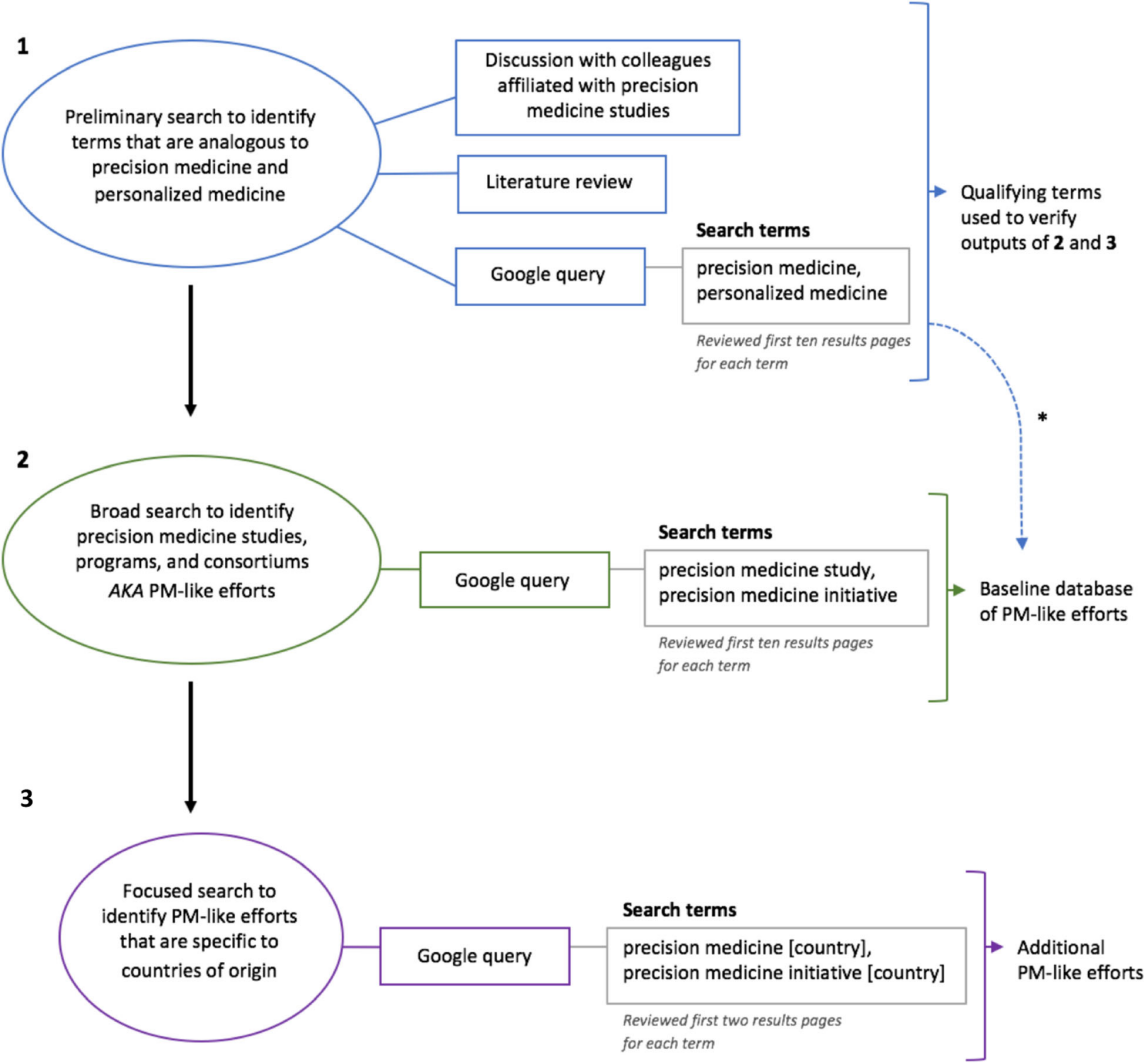
Outline of our exploratory methods to: 1. gather and define the terminology used, analogous with precision medicine, 2. compile a database of precision medicine efforts, and 3. account for global precision medicine efforts *Results from the preliminary search fed into our baseline database of PM-like efforts

Formative and exploratory research was conducted to better understand the nature and scope of programs described as a “precision medicine” initiative, study or program. The chronology of our fluid, predominantly web-based search is outlined in Fig. 5.

### Identifying relevant keywords

A first step was to identify words that may be used synonymously with a precision medicine initiative or study. These analogous terms were identified via discussions with colleagues affiliated with large scale research data resource development initiatives (e.g., NIH AoURP, Humans of New York University), a review of relevant literature, and a Google query of “(precision *or* personalized) medicine.” The online search was intentionally capped at the first ten results pages, each page containing about ten results.

### Resulting precision medicine analogous terms

This process resulted in a total of nine analogous terms that were used as qualifying terms to identify a PM-like effort to include in our database (see Table 3). Besides “precision medicine” and “personalized medicine,” which have no functional difference, “genomic medicine,” “individualized medicine,” “evidenced-based medicine,” “stratified medicine,” “big data,” “translational medicine,” and “P4 medicine i.e. predictive, preventive, personalized and participatory” medicine were added. The peer reviewed literature was consulted to provide formal definitions for these analogous terms (see Table 3).

**Table 3:** Qualifying terms analogous to precision medicine with verbatim definitions

Initial search terms	Precision *or* personalized medicine (initiative *or* study)
	Precision medicine *and* personalized medicine: tailoring of medical treatment to the individual characteristics of each patient … the ability to classify individuals into subpopulations that differ in their susceptibility to a particular disease, in the biology and/or prognosis of those diseases they may develop, or in their response to a specific treatment … [2]
	Personalized medicine: improvements in the stratification and timing of health care by utilizing biological information and biomarkers on the level of molecular disease pathways, genetics, proteomics, and metabolomics [29]
Secondary search terms	Precision medicine (initiative) [country]
Terms discovered through literature that often relate to or are used in place of precision medicine	Genomic medicine: clinical care based on genomic information [30]
	Individualized medicine: medicine that is particularized to a human being [31]
	Evidence-based medicine: conscientious, explicit, and judicious use of current best evidence in making decisions about the care of individual patients [32]
	Stratified medicine: approach of proactively testing and selecting populations for specific treatments [33]
	Big data: high-volume, high-velocity and/or high-variety information assets that enable enhanced insight, decision making, and process automation [34]
	Translational medicine: an interdisciplinary branch of the biomedical field supported by three main pillars: benchside, bedside, and the community; the goal is to combine disciplines, resources, expertise, and techniques within these pillars to promote enhancements in prevention, diagnosis, and therapies [35]
	P4 medicine: a systems approach to medicine that includes predictive, personalized, preventive, and participatory aspects; a revolution that is fueled by an appreciation for medicine as an information science, a holistic approach to studying the complexities of disease, emerging technologies that allow us to explore new dimensions of patient data space, and powerful new analytic technologies [36]

### Compiling PM-like efforts

A Google query was conducted to compile a database of potential programs, studies, and consortiums that use precision medicine-like terminology, which were labeled “PM-like efforts.” We used the search term schematic “precision medicine (initiative or study).” The qualifier of “study” or “initiative” was appended to avoid results that would only yield descriptions of precision medicine. The first ten results pages returned for each phrase, containing about ten results, were reviewed for potential PM-like efforts. Any public-facing online content, from project websites and popular articles to uploaded protocol documents, were reviewed to verify that they were either claiming to be a precision medicine endeavor, or they were being portrayed as one. If a program was mentioned by one of the directly linked websites and no other links on the website’s pages were provided, the program was specifically searched further to collect more information. Literature found via web search was used to augment our results, so a PM-like effort may have been mentioned and/or described exclusively in a news or journal article. Each PM-like effort included in our database required a purpose inclusive of one or more of the qualifying terms in Table 3, either directly on the project’s website or in the referencing article. One-time entities, such as a genetic test being offered at a hospital or by a genetic testing company, were not included as PM-efforts; neither were workshops, conferences, seminars, or symposiums. This exploratory search was systematic, to the extent possible, but due to the lacking consistency of program descriptions and the wide variability in programs utilizing precision medicine terminology, the process of deciphering whether a program would be added to our database was somewhat subjective; albeit, based on a thorough review of the available data.

### Global representation

The results of this initial search and review of relevant literature, such as the World Innovation Summit for Health (WISH) Forum Report of 2016, [19] prompted the research team to question whether the initial search terms were inclusive of global efforts. To ensure the list was inclusive of global programs, the search term schematic was expanded to include “precision medicine (initiative) [country],” where the “country” was pulled from lists of the fifty most populous and wealthiest countries created by Central Intelligence Agency World Factbook [37] and Global Finance [38], respectively. This filter was applied to broaden our search and account for domestic bias, and to test whether PM-like efforts mapped to countries with ample funding and population size, which are often crucial for large cohort studies. The first two results pages, with approximately ten results per page, of this secondary search were reviewed and coded. Fewer results pages were reviewed for the global query when compared to our initial search, because including the country name made the search much more specific, and arguably the most popularized PM-like efforts, if any, would be mentioned within the first few results.

### Codifying and mapping

The open-ended nature of Google querying was necessary to identify descriptive variables and to develop a codebook of terms or phrases qualifying a PM-like effort and its characteristics. For each PM-like effort, its descriptive characteristics were entered into the Excel database. Variables included geographic location, duration, data type(s), funding entities, study aim(s), sponsor definition of “precision medicine” or its equivalent, sample size, and any demographic or disease-related attributes (see Table 4). For example, per program or study, the data types it involved were coded as one or more of the following: biospecimen collection, electronic health records (EHR), sensor data, personal provided information (PPI), and existing databases. All web-based searches were conducted over a one-month period (June 19, 2017 through July 20, 2017).

**Table 4:** Codebook of variables used to characterize PM-like efforts

Theme	Category	Qualifying words or phrases (when applicable)
Longitudinal	Yes	“longitudinal,” “cohort”
	No	
Data type	Biospecimen	“blood,” “saliva,” “urine,” “genome,” “genetic screening,” “biomarkers,” “laboratory results”
	Electronic Health Record (EHR)	“electronic health record,” “electronic medical record,” “patient registry,” “clinical outcomes,” “e-health,” mention of recruitment through hospital
	Personal Provided Information (PPI)	Mention of questionnaire or survey about environmental, behavioral, and/or lifestyle factors
	Sensor	“wearable/smart technology/mobile sensor,” “(daily) monitoring,” “physiological data”
Funding	Government	mention of government body or subsidiary e.g. “national institute,” “ministry,” “parliament,” “council,” and “public research university”
	Industry	
	Nonprofit	
	Donations	
Study Aim	Database	mention of building a platform or baseline, combining genomic and [phenotypic data] or [medical information], “data storage,” “biobank,” “registry,” “repository”
	Clinical Trial	mention of clinical application or intervention, “demonstration projects”
	Review	consolidated funding entities, meta-analyses of existing databases, mention of a consortium or center that forms partnerships and supports various projects in applied and basic research, policy-making, and data analytics i.e. programs that “review” and support progress in an overarching way
Patients/Participants considered partners	Yes	“need you,” “get involved,” “patient-centered,” call-to-action language in enrollment, return of results
	No	

### Data analysis

Qualitative methods were used to develop codes that were then quantified using descriptive statistics. Frequencies were calculated to quantify the types of data collected, funding sources, underlying objectives, and cohort scope. Geographic distributions of the PM-like efforts were created using BatchGeo (http://batchgeo.com). These data were then organized across a spectrum and compared with benchmark characteristics of the AoURP for context. Specifically, programs were coded to denote whether they included a multidimensional dataset, a sample size greater than 10,000 participants, and a longitudinal design.

## Data Availability

Data are available and can be appended.

## Abbreviations

AoURP: All of Us Research Program
EHR: Electronic Health Records
NIH: National Institutes of Health
NRC: National Research Council
PMI: Precision Medicine Initiative
PPI: Personal Provided Information
WISH: World Innovation Summit for Health

## References

[1] Collins FS, Varmus H (2015). A new initiative on precision medicine. New Eng J Med.

[2] Toward Precision Medicine: Building a Knowledge Network for Biomedical Research and a New Taxonomy of Disease. In: The National Academies Press. National Research Council. 2011. https://www.nap.edu/catalog/13284/toward-precision-medicine-building-a-knowledge-network-for-biomedical-research. Accessed 20 June 2017. 22536618

[3] Remarks by the President on Precision Medicine. In: The White House President Barack Obama. Office of Press Secretary. 2015. obamawhitehouse.archives.gov/the-press-office/2015/01/30/remarks-president-precision-medicine. Accessed 1 Aug 2017.

[4] All of Us Research Program Operational Protocol. National Institutes of Health. 2018. https://allofus.nih.gov/sites/default/files/aou_operational_protocol_v1.7_mar_2018.pdf. Accessed 19 June 2017.

[5] Chavarro JE, Rich-Edwards JW, Gaskins AJ (2016). Contributions of the nurses’ health studies to reproductive Health Research. Am J Public Health.

[6] Mahmood SS, Levy D, Vasan RS (2014). The Framingham heart study and the epidemiology of cardiovascular disease: a historical perspective. Lancet.

[7] Hawgood S, Hook-Barnard IG, O’Brien TC (2015). Precision medicine: Beyond the inflection point. Sci Transl Med.

[8] Feldman AM (2015). Bench-to-bedside; clinical and translational research; personalized medicine; precision medicine-What’s in a name?. Clin Transl Sci.

[9] Beckmann JS, Lew D. Reconciling evidence-based medicine and precision medicine in the era of big data: challenges and opportunities. Genome Med. 2016;8(1):134. 10.1186/s13073-016-0388-7 PMC5165712 27993174

[10] Bayer R, Galea S (2015). Public health in the precision-medicine era. New Eng J Med.

[11] Vaithinathan AG, Asokan V (2017). Public health and precision medicine share a goal. J Evid Based Med.

[12] Parikh RB, Schwartz JS, Navathe AS (2017). Beyond genes and molecules — a precision delivery initiative for precision medicine. New Eng J Med.

[13] Riley WT, Nilsen WJ, Manolio TA (2015). News from the NIH: potential contributions of the behavioral and social sciences to the precision medicine initiative. Transl Behav Med.

[14] Scudellari M (2017). Big science has a buzzword problem. Nature..

[15] Mirnezami R, Nicholson J, Darzi A (2012). Preparing for precision medicine. New Eng J Med.

[16] Kohane IS (2015). Ten things we have to do to achieve precision medicine. Science.

[17] Desmond-Hellmann S (2012). Toward Precision Medicine: A New Social Contract?. Sci Transl Med.

[18] Hudson KL, Collins FS (2015). Bringing the common rule into the 21st century. New Eng J Med.

[19] Dzau V, Ginsburg GS, Finkelman E, et al. Precision Medicine A Global Action Plan for Impact. In: WISH Forums Reports: World Innovation Summit for Health; 2016. https://www.wish.org.qa/wp-content/uploads/2018/01/IMPJ4495_WISH_Precision_Medicine_Report_WEB.pdf. Accessed 1 July 2017.

[20] Manolio TA, Abramowicz M, Al-mulla F (2016). Global implementation of genomic medicine: we are not alone. Sci Transl Med.

[21] Innovate UK: Mapping the UK Precision Medicine Landscape. 2015. https://assets.publishing.service.gov.uk/government/uploads/system/uploads/attachment_data/file/483560/Precision_Medicines_Booklet_Final_Web__002_.pdf. Accessed 23 June 2017.

[22] Lu J, Xuan S, Downing NS (2016). Protocol for the China PEACE (patient-centered evaluative assessment of cardiac events) million persons project pilot. BMJ Open.

[23] InnVentis: Platform. 2015. http://www.innventis-pharma.com/platform.php. Accessed 18 July 2019.

[24] Ferryman K, Pitcan M. Fairness in Precision Medicine: Data & Society; 2018. https://datasociety.net/output/fairness-in-precision-medicine/. Accessed 30 Mar 2018.

[25] Chowkwanyun M, Bayer R, Galea S (2018). “Precision”Public Health – Between Novelty and Hype. New Eng J Med.

[26] Kravitz RL, Duan N (2014). Eds, and the DEcIDE methods center N-of-1 guidance panel (Duan N, Eslick I, Gabler NB, Kaplan HC, Kravitz RL, Larson EB, pace WD, Schmid CH, Sim I, Vohra S). Design and implementation of N-of-1 trials: a User’s guide. AHRQ publication no. 13(14)-EHC122-EF.

[27] Hunter DJ (2016). Uncertainty in the era of precision medicine. New Eng J Med.

[28] Bloss C, Nebeker C, Bietz M (2016). Reimagining human research protections for 21st century science. J Med Internet Res.

[29] Schleidgen S, Klingler C, Bertram T, Rogowski WH, Marckmann G (2013). What is personalized medicine: sharpening a vague term based on a systematic literature review. BMC Med Ethics.

[30] Green ED, Guyer MS (2011). Charting a course for genomic medicine from base pairs to bedside. Nature.

[31] Topol EJ (2014). Individualized medicine from Prewomb to tomb. Cell.

[32] Sackett DL, Rosenberg WM, Gray JA, Haynes RB, Richardson WS (1996). Evidence based medicine: what it is and what it isn’t. BMJ.

[33] Trusheim MR, Berndt ER, Douglas FL (2007). Stratified medicine: strategic and economic implications of combining drugs and clinical biomarkers. Nat Rev Drug Discov.

[34] Big Data. In: IT Glossary. Gartner, Inc. 2019. https://www.gartner.com/it-glossary/big-data/. Accessed 27 Feb 2019.

[35] Cohrs RJ, Martin T, Ghahramani P, Bidaut L, Higgins PJ, Shahzad A (2015). Translational medicine definition by the European Society for Translational Medicine. New Horiz Transl Med.

[36] Hood L, Friend SH (2011). Predictive, personalized, preventive, participatory (P4) cancer medicine. Nat Rev Clin Oncol.

[37] Central Intelligence Agency World Factbook: Country Comparison: Population. 2017. https://www.cia.gov/library/publications/resources/the-world-factbook/rankorder/2119rank.html. Accessed 3 July 2017.

[38] Ventura L. Global Finance: Richest Countries in the World 2017. 2017. https://www.gfmag.com/global-data/economic-data/richest-countries-in-the-world?page=12. Accessed 3 July 2017.

